# Pyridazine-bridged cationic diiridium complexes as potential dual-mode bioimaging probes†

**DOI:** 10.1039/c8ra00265g

**Published:** 2018-03-06

**Authors:** Ruth E. Daniels, Luke K. McKenzie, Jonathan R. Shewring, Julia A. Weinstein, Valery N. Kozhevnikov, Helen E. Bryant

**Affiliations:** Department of Applied Sciences, Faculty of Health and Life Sciences, Northumbria University Tyne and Wear NE1 8ST Newcastle Upon Tyne UK valery.kozhevnikov@northumbria.ac.uk +44 (0) 191 243 7430; Department of Chemistry, University of Sheffield Dainton Building Sheffield S3 7HF UK; Academic Unit of Molecular Oncology, Sheffield Institute for Nucleic Acids (SInFoNiA), Department of Oncology and Metabolism, University of Sheffield Beech Hill Road Sheffield S10 2RX UK h.bryant@sheffield.ac.uk +44 (0) 114 2795320 +44 (0) 114 2759040

## Abstract

A novel diiridium complex [(N^C^N)_2_Ir(bis-N^C)Ir(N^C^N)_2_Cl]PF_6_ (N^C^N = 2-[3-*tert*-butyl-5-(pyridin-2-yl)phenyl]pyridine; bis-N^C = 3,6-bis(4-*tert*-butylphenyl)pyridazine) was designed, synthesised and characterised. The key feature of the complex is the bridging pyridazine ligand which brings two cyclometallated Ir(iii) metal centres close together so that Cl also acts as a bridging ligand leading to a cationic complex. The ionic nature of the complex offers a possibility of improving solubility in water. The complex displays broad emission in the red region (*λ*_em_ = 520–720 nm, *τ* = 1.89 μs, *Φ*_em_ = 62% in degassed acetonitrile). Cellular assays by multiphoton (*λ*_ex_ = 800 nm) and confocal (*λ*_ex_ = 405 nm) microscopy demonstrate that the complex enters cells and localises to the mitochondria, demonstrating cell permeability. Further, an appreciable yield of singlet oxygen generation (*Φ*_Δ_ = 0.45, direct method, by ^1^O_2_ NIR emission in air equilibrated acetonitrile) suggests a possible future use in photodynamic therapy. However, the complex has relatively high dark toxicity (LD_50_ = 4.46 μM), which will likely hinder its clinical application. Despite this toxicity, the broad emission spectrum of the complex and high emission yield observed suggest a possible future use of this class of compound in emission bioimaging. The presence of two heavy atoms also increases the scattering of electrons, supporting potential future applications as a dual fluorescence and electron microscopy probe.

## Abstract 2

A novel cell permeable, mitochondria localising, diiridium complex has a high emission yield and two heavy atoms to increase scattering of electrons, supporting potential future applications as a dual fluorescence and electron microscopy probe.

## Graphical abstract

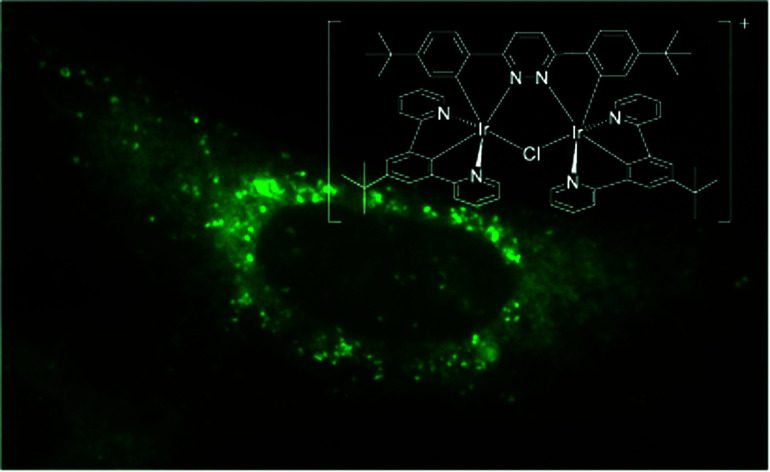

## 1. Introduction

In comparison to traditional bio-imaging and photodynamic therapy (PDT) agents, transition metal complexes combine longer emission lifetimes and higher photo-stability, with relative ease of chemical modification.^1^ During imaging, longer emission lifetimes permit the use of time-gating to reduce auto-fluorescence and can be utilised to detect biologically relevant analytes, such as O_2_*in vitro*^1,2^ and *in vivo*^3^, while in PDT high emission lifetimes lead to high yields of ^1^O_2_ and/or other ROS, and thus efficient photo-induced cell killing. Increased photostability favours longer term tracking during bio-imaging,^4,5^ and increases the photosensitizing activity of an agent during PDT.

In the field of transition metal complexes for use in bio-imaging, ruthenium, rhenium and platinum complexes dominated the early literature. However, iridium complexes with high emission quantum yields and localisation to a wide variety of sub-cellular organelles have seen a surge in popularity over the last decade.^6–11^ In PDT, ruthenium complexes have dominated the field of transition metal photosensitizers^12–16^ with one Ru(ii) complex having entered clinical trials in Canada.^17,18^ In comparison, iridium complexes in PDT are yet to be fully explored. Importantly a few iridium complexes have been recently shown to have photo-toxicities at low light doses under both one-^19–25^ and two-photon excitation,^26–30^ suggesting that Ir(iii) containing complexes hold future potential for PDT.

Previously we reported several families of highly luminescent transition metal (TM) complexes linked by cyclometallating ditopic ligands.^31–34^ In comparison with their monometallic analogues, these polynuclear complexes show red-shifted absorption and luminescence properties that may minimise the background auto-fluorescence in imaging of biological objects. In addition, the appreciable molar extinction coefficient of the low-energy absorption band and high quantum yields make them good imaging agents. The red-shift of the absorption band is also advantageous for PDT because it allows for excitation by wavelengths of light with greater tissue depth penetration. The highest quantum yields we previously observed with any of the complexes (up to 100%) were for the di-iridium(iii) complexes formed by ditopic bis-N^C ligands in conjunction with symmetrically-substituted terminal N^C^N ligands.^35^ These complexes were also the easiest to synthesise because the iridium centres are achiral and the formation of diastereomers is not possible; the strong *trans* influence of the metallated rings of both the N^C^N and bis-N^C ligands directs the relative positions of these ligands around the iridium centres so that only one product is formed.^35^ The presence of two closely positioned heavy atoms might also increase the scattering of electrons suggesting the use of di-iridium complexes as contrast reagents and thus combined with the high quantum yields as dual-modality agents. However, all the complexes prepared so far were designed for applications in organic light emitting diodes and none are soluble in water.

Here, motivated by the advantages offered by bimetallic Ir(iii) complexes, we sought to adapt the complexes by incorporating pyridazine as a bridging heterocycle. The close proximity of Ir atoms causes the Cl atom to act as a bridging ligand. Five chelate rings form as a result, providing the driving force for the reaction and leading to a rigid architecture. Another consequence of the bridging Cl atom is that the complex becomes ionic, which somewhat improves solubility in water. Once synthesised, we characterised the complex and examined its ability to enter cells, its sub-cellular localisation and its cellular toxicity. In addition, it's use as a contrasting reagent in transmission electron microscopy (TEM) was assessed.

## 2. Results and discussion

### 2.1. Synthesis

**Fig. 1:**
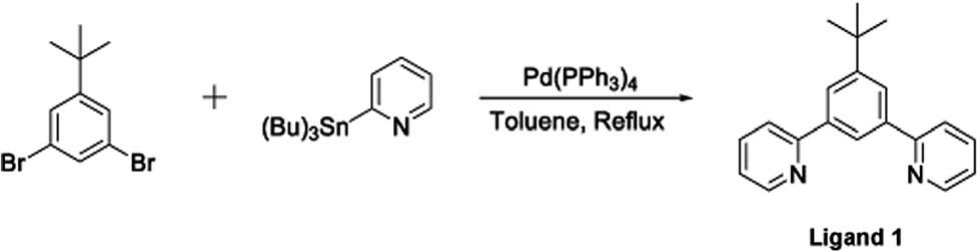
Synthesis of the terdentate proligand (Ligand 1) *via* a Stille cross-coupling reaction.

The terdentate proligand, 2-[3-*tert*-butyl-5-(pyridin-2-yl)phenyl]pyridine (Ligand 1), was prepared using Stille cross-coupling methodology (Fig. 1) and was isolated in good yield. Chou *et al.* previously reported the synthesis of this terdentate ligand *via* an alternative Suzuki–Miyaura cross-coupling route. The inclusion of a *tert*-butyl substituent at the 5-position of the central phenyl ring ensures terdentate coordination to an iridium(iii) metal centre.^36^

**Fig. 2:**
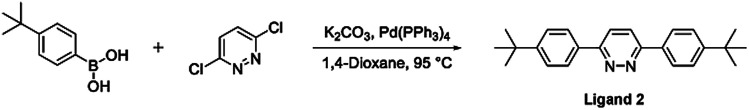
Synthesis of the bis-N^C coordinating bridging ligand (Ligand 2) *via* a Suzuki–Miyaura cross-coupling reaction.

The synthesis of the bis-N^C coordinating proligand, 3,6-bis(4-*tert*-butylphenyl)pyridazine (Ligand 2), was achieved through a Suzuki–Miyaura cross-coupling reaction between 3,6-dichloropyridazine and 4-(*tert*-butyl)phenylboronic acid (Fig. 2). The desired product was isolated in good yield. A similar synthetic route for the preparation of 3,6-bis(4-*tert*-butylphenyl)pyridazine was previously published by Wang and co-workers.^37^

**Fig. 3:**
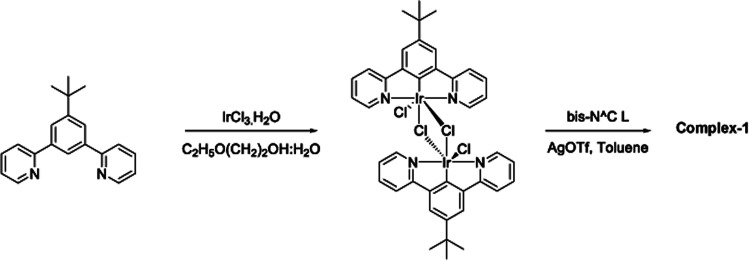
Synthesis of [(N^C^N)_2_Ir(bis-N^C)Ir(N^C^N)_2_Cl]PF_6_ (N^C^*N* = 2-[3-*tert*-butyl-5-(pyridin-2-yl)phenyl]pyridine; bis-N^C = 3,6-bis(4-*tert*-butylphenyl)pyridazine) (complex-1) by reaction of dichloro-bridged iridium(iii) dimer with the bis-bidentate proligand in toluene, using silver triflate as a chloride scavenger.

**Fig. 4:**
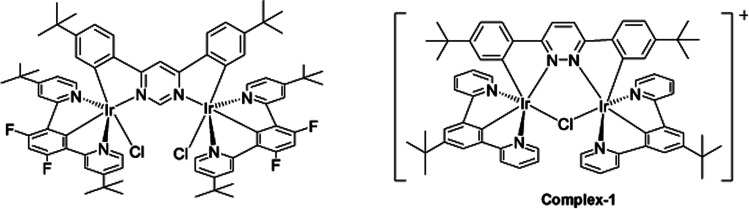
Changing bridging heterocycle from pyrimidine to pyridazine leads to cationic di-Ir complex (complex-1).

The target dinuclear complex was prepared using a similar synthetic method to that developed by Williams.^35^ Firstly, the terdentate Ligand 1 was reacted with IrCl_3_·H_2_O at reflux in a mixture of 2-ethoxyethanol and water to give the dichloro-bridged iridium dimer, [Ir(N^C^N)Cl(μ-Cl)]_2_. in 85% yield. The dimer was then reacted with the bis-bidentate proligand in toluene, using silver triflate as a chloride scavenger (Fig. 3). Upon completion of the reaction, the mixture was treated with excess HCl (3 M, aq.) in order to ensure that the only monodentate ligand present on an iridium centre is chloride. Following this, a Cl/PF_6_ anion exchange was carried out using a saturated solution of KPF_6_(aq). Purification was carried out using column chromatography to give the target complex 1 (Fig. 4), in 38% yield.^35^ The complex was characterised by ^1^H and spectroscopy and high resolution mass spectrometry (ESI S3†).

### 2.2. Photophysical studies

In acetonitrile solution, 1 shows broad absorbtion in the UV/vis region with appreciable absorption up to ∼500 nm. Upon excitation (*λ*_exc_ = 400 nm) 1 shows broad emission (*λ*_em_ = ∼520–720 nm). An emission lifetime of 1.9 μs and a quantum yield of 62% (±11%) was measured in deaerated (by bubbling N_2_) acetonitrile solution. Emission q.y. decreased ∼10-fold, to 5%, in aerated solution indicating significant quenching of the emissive state by oxygen. The singlet oxygen yield, *Φ*_Δ_ = 0.45 was measured by direct detection of the ^1^O_2_ emission (1270 nm) under UV excitation (*λ*_exc_ = 355 nm). The appreciable singlet oxygen yield showed the potential for this class of compounds as therapeutic agents (Table 1 and ESI S1†).

**Table 1:** Summary of the photophysical data for 1

Compound	*λ*_max_ a [nm] (*ε* [× dm^3^ mol^−1^ cm^−1^])	*λ*_em_ a ^,^ b (max)	*τ*_0_, *τ*_1_, *τ*_2_^a^^,^^c^ [ns]	*Φ*_0_, *Φ*_1_^a^^,^^b^^,^^d^ [%]	*Φ*_Δ_ a ^,^ e [%]
1	235 (79 900), 284 (54 700), 397 (24 200)	576	1890, 226, 561/103	62, 5	45

a Acetonitrile.

b *λ*_exc_ = 400 nm.

c *τ*_0_ = degassed acetonitrile, *τ*_1_ = acetonitrile, *τ*_2_ = water from DMSO stock.

d *Φ*_0_ = degassed, *Φ*_1_ = aerated [Ru(Bipy)_3_](PF_6_)_2_ standard.

e *λ*_exc_ = 355 nm, perinapthenone standard.

### 2.3. Luminescent imaging and sub-cellular localisation

The complex is moderately soluble in water, DMSO was used to assist solubilisation with the final concentration of DMSO always ≤2%.

**Fig. 5:**
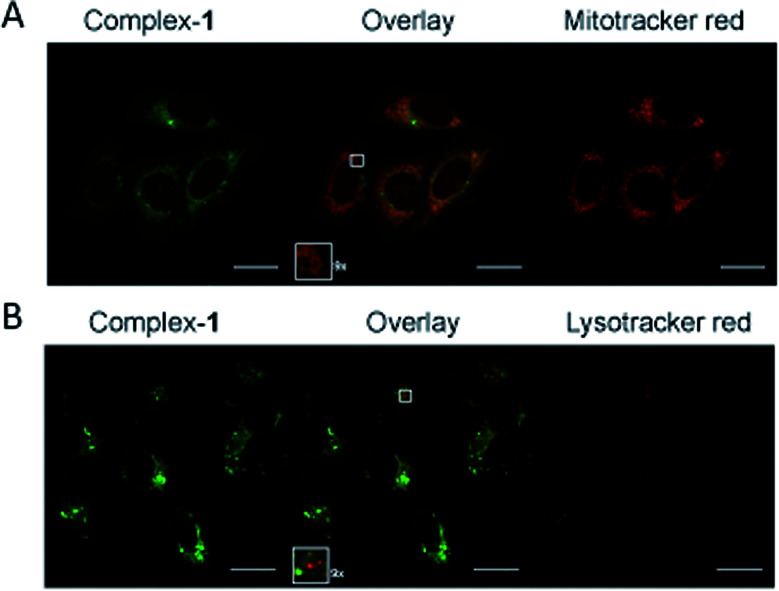
Sub-cellular localisation of complex-1. HeLa cells following 5 hour incubation with 10 μM complex-1 (green), colocalised with (A) Mitotracker orange (red) or (B) Lysotracker (red). Zoomed sections (×9) are shown as insets. Scale bars = 20 μm.

Emission microscopy of live cells incubated with 10 μM 1, performed under multiphoton excitation (*λ*_exc_ = 800 nm), showed that 1 enters cells and localises in the cytoplasm with distinct punctate staining (ESI S2†). These results are consistent with those reported previously for cationic metal complexes, which are known to locate in mitochondrial and lysosomal structures.^38^ Co-localisation of 1 with mitochondria was confirmed using organelle specific stains and confocal microscopy (*λ*_exc_ = 405 nm) (Fig. 5A), giving a Pearson's correlation coefficient of *R* = 0.76 (averaged over 15 cells) for mitotracker red. Co-localisation of 1 with lysotracker red was not observed (Fig. 5B) indicating that the punctate staining may result from other endosomal structures (Pearson's correlation coefficient of *R* = 0.53 with lysotracker red (averaged over 15 cells)).

### 2.4. Cellular toxicity

**Fig. 6:**
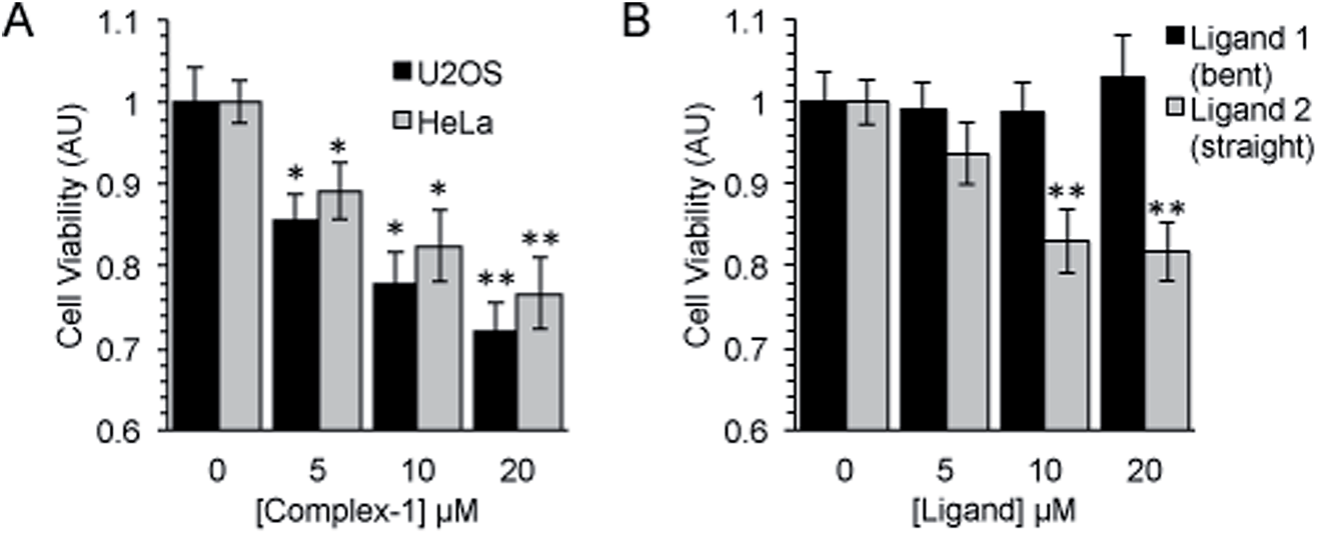
Complex-1 and Ligand 2 induce a significant reduction in cell viability. Cell viability as measured by MTT assay after a 5 hour incubation with (A). Complex-1 in HeLa and U2OS cells and (B). Ligands as indicated in HeLa cells. Mean and SEM of 4 independent repeats is shown. Significance calculated by Student's *T*-test compared to respective untreated control is indicated, where * = *p* < 0.05 and ** = *p* < 0.01.

**Fig. 7:**
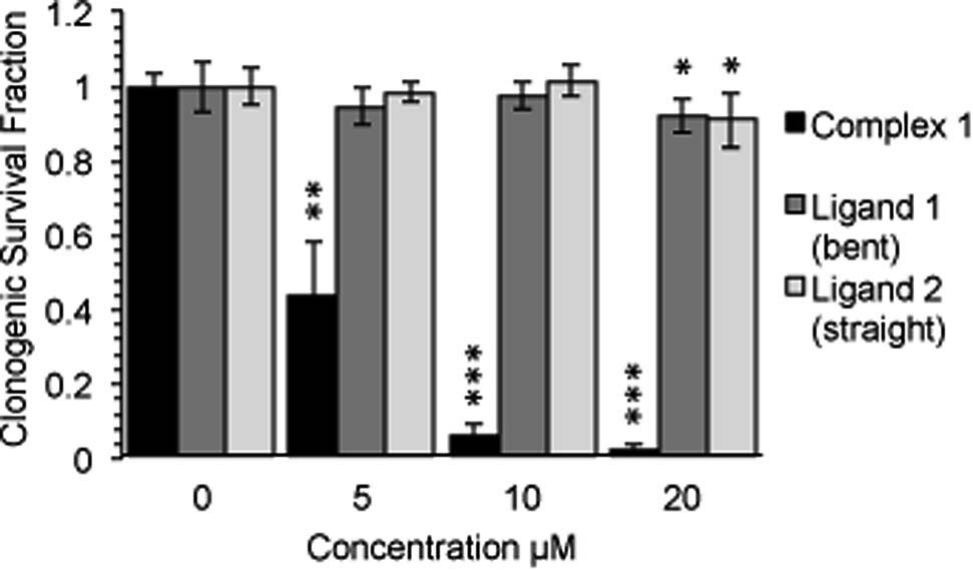
Complex-1 reduces cell survival to a significantly greater degree than each ligand alone. Long term survival (10 days) as measured by clonogenic survival assay after a 5 hour incubation with complex-1/Ligands as indicated in HeLa cells. Mean and SD of 2 independent repeats (each conducted in duplicate is shown). Significance calculated by Student's *T*-test compared to respective untreated control is indicated, where * = *p* < 0.05, ** = *p* < 0.01 and *** = *p* < 0.001.

Following an incubation time of 5 hours to mimic imaging conditions, 1 induced a significant dose dependent reduction in metabolic activity/cell viability in both U2OS and HeLa cancer cell lines as indicated by MTT assay (Fig. 6A). The effect of the associated ligands was also measured. Ligand 1, did not reduce cell viability whilst Ligand 2, induced a similar reduction in metabolic activity as 1 (Fig. 6B). The MTT assay measures the metabolic activity of a cell, and thus is considered a read out of cell viability, however it is not a measure of long-term cell survival. A clonogenic survival assay was used in order to obtain an assessment of the cellular toxicity. In these assays, cells were plated at low density prior to a 5 hour incubation with 1 or its associated ligands, subsequently cells were left to recover and form colonies for 10 days. In this more reliable assay of cell toxicity 1 showed significant toxicity (LD_50_ = 4.46 μM) while the associated ligands induced only slight toxicity at the highest doses tested (LD_50_ ≥ 20 μM) (Fig. 7). Taken together the MTT and clonogenic survival data reveal that while **Ligand 2** transiently effects the metabolic activity of cells, over the short term 1 is significantly more cytotoxic to cells than either of the ligands.

### 2.5. Transmission electron microscopy (TEM)

**Fig. 8:**
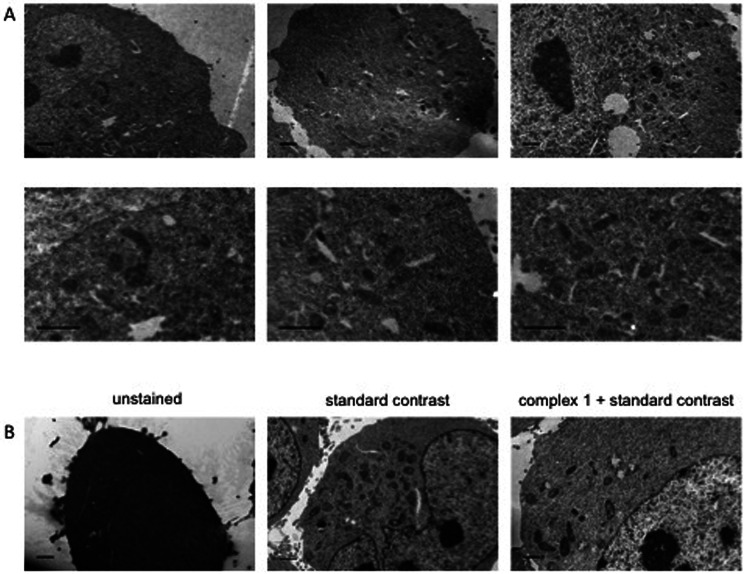
Transmission Electron Microscopy images of HeLa cells using complex-1 as contrast agent. Cells incubated with or without 20 μM complex-1 for 4 h, example images with and without standard contrast (“double contrasting”, with uranyl acetate and lead citrate). (A) Example images of staining with complex-1 alone. (B) Example images of cells unstained, stained using standard double contrast agents and stained with complex-1 plus standard agents. Scale bars = 1 μm.

Dual-modality imaging agents have district advantages over agents used in a single method, combining strengths of individual imaging techniques. The potential of 1 as a staining agent in transmission electron microscopy (TEM) was evaluated as follows. Live U2OS cells were incubated with 1 (20 μM, 4 hours), fixed, processed for TEM in the presence or absence of standard contrasting agents, and imaged (Fig. 8). Contrast in the cells stained with 1 was enhanced compared to unstained cells. Interestingly 1 also increased contrast in organelles other than mitochondria, this raises the possibility that the microenvironment in live cells means that luminescence is greater in the mitochondria, alternatively the complex might leak and diffuse around the cell while being prepped for TEM as we didn't use any OsO_4_ which acts as a secondary fixative. Importantly, 1 did not interfere with standard contrast enhancing reagents (uranyl acetate and lead citrate).

## 3. Conclusion

We have demonstrated that a novel pyridazine-bridged cationic di-iridium complex can enter live cells, has photo-physical properties favourable for imaging applications, and generates singlet oxygen in considerable yield. However, due to high dark cytotoxicity, this complex is unlikely to be suitable for PDT applications. Instead, the toxicity of the complex could be exploited in other applications where its intrinsic toxicity could be beneficial, such as development of new anticancer agents. The photophysical properties of the complexes are of interest for luminescent bioimaging and the presence of two heavy atoms allows its use as a contrast agent in TEM. Potentially, the structure of the complex can be varied trough modification of both bridging and auxiliary ligands as well as by employing different metals to fine-tune the properties and reduce toxicity.

## 4. Materials and methods/experimental section

### 4.1. Synthesis and characterisation

#### 4.1.1. Ligand 1^36^

A mixture of 1,3-dibromo-5-(*tert*-butyl)benzene (3.01 g, 10.3 mmol), 2-(tributylstannyl)pyridine (9.54 g, 25.7 mmol) and tetrakis(triphenylphosphine)palladium (0.358 g, 0.309 mmol) were added to toluene (100 mL) and the reaction mixture degassed for 15 min under argon. The reaction mixture was heated to reflux (140 °C) for 20 h. The reaction mixture was purified by column chromatography (silica gel, petroleum ether : ethyl acetate 100 : 0 to 4 : 1 to 6 : 1 to 3 : 1) and a white solid was obtained (2.11 g, 7.31 mmol, 71%). ^1^H NMR (400 MHz, CDCl_3_): *δ* 8.72 (d, 2H, *J* = 1.6), 8.34 (d, 1H, *J* = 1.4), 8.12 (d, 2H, *J* = 1.4), 7.82 (d, 2H, *J* = 7.8), 7.76 (t, 2H, *J* = 7.8), 7.26–7.23 (m, 2H), 1.45 (s, 9H); ^13^C NMR (CDCl_3_, 100 MHz): *δ* 157.83 (C), 152.23 (C), 149.66 (CH), 139.71 (C), 136.67 (CH), 124.75 (CH), 123.02 (CH), 122.08 (CH), 120.93 (CH), 35.12 (C), 31.48 (C–(CH_3_)_3_).

#### 4.1.2. Ligand 2^37^

3,6-Dichloropyridazine (0.497 g, 3.33 mmol), (4-*tert*-butylphenyl)boronic acid (1.54 g, 8.67 mmol) and a 2 M aqueous potassium carbonate solution (2.76 g, 20.0 mmol, 10.0 mL) were added to a round-bottomed flask. 1,4-Dioxane (25 mL) was added. The mixture was degassed under argon for 20 min. Tetrakis(triphenylphosphine)palladium (0.231 g, 0.200 mmol) was added and the reaction mixture degassed for a further 10 minutes. The reaction was heated to 95 °C for 42 h. The reaction was cooled to room temperature. A precipitate formed and this precipitate was filtered *in vacuo*. The solid was then recrystallised from dimethylformamide to give the product as a silver solid (0.659 g, 1.91 mmol, 57%). ^1^H NMR (CDCl_3_, 400 MHz): *δ* 8.11 (d, 4H, *J* = 8.2), 7.90 (s, 2H), 7.57 (d, 4H, *J* = 8.2), 1.39 (s, 18H); ^13^C DEPT135 NMR (CDCl_3_, 400 MHz): *δ* 157.24 (C), 153.28 (C), 133.33 (C), 126.58 (C–H), 126.02 (CH), 123.86 (CH), 34.83 (C), 31.26 (C–(CH_3_)_3_).

#### 4.1.3. 1

##### Step 1

A mixture of 1,3-di(2-pyridyl)-5-(*tert*-butyl)benzene Ligand 1 (0.609 g, 2.08 mmol) and iridium chloride hydrate (0.768 g, 2.08 mmol) were added to a 3 : 1 mixture of 2-ethoxyethanol and water (100 mL). The reaction mixture was heated to reflux (bath temperature 130 °C) for 24 h under argon and then allowed to cool to room temperature. The resulting orange solid was filtered off, washed with ethanol and water to give intermediate dichlorobridged iridium complex (0.972 g, 1.77 mmol, 85%) which was used in the following step without further purification.

##### Step 2

The dichlorobridged iridium complex from step 1 (0.170 g, 0.308 mmol) was added to a round-bottomed flask. Toluene (30 mL) was added and the mixture stirred. Silver triflate (0.122 g, 0.463 mmol) was added. 3,6-Bis(4-*tert*-butylphenyl)pyridazine Ligand 2 (0.0531 g, 0.154 mmol) was added and the reaction mixture was heated to reflux for 15 h. The reaction mixture was removed from the heat and 3 M hydrochloric acid solution (7 mL) was added. The mixture was stirred for 5 min. The solvent was evaporated to dryness. A mixture of 1 : 1 acetonitrile : water (4 mL) was added to the residue. To the solution formed, excess saturated aqueous ammonium hexafluorophosphate solution was added. An orange solid precipitated and was filtered *in vacuo* to give the crude product (0.233 g, 0.174 mmol). The product was then purified by column chromatography (silica gel, DCM : EtOAc 10 : 1). Yellow solid (0.0781 g, 0.0584 mmol, 38%). ^1^H NMR (CDCl_3_, 400 MHz): *δ* 8.72 (s, 2H), 8.00 (d, 4H, *J* = 5.0), 7.71 (dd, 6H, *J* = 8.2, 3.2), 7.689 (s, 4H), 7.50 (td, 4H, *J* = 7.3, 1.8), 6.94–6.92 (m, 6H), 6.00 (d, 2H, *J* = 1.8), 1.37 (s, 18H), 0.87 (s, 18H). HRMS (FTMS^+^) for [M]^+^ calcd. 1337.4129, found 1337.4144; for [M–(Ir(NCN))–Cl]^+^ calcd. 823.0780, found 823.3359.

### 4.2. Photophysical studies

The UV/vis spectra were recorded on Varian Cary 5000 UV-vis-NIR spectrophotometer in 1 cm quartz cuvettes. The luminescence spectra were recorded on Fluoromax 4 spectrofluorimeter (HORIBA Jobin Yvon). Emission lifetime measurements were performed on the mini-τ spectrofluorometer (Edinburgh Instruments), with 405 nm pulsed diode laser as an excitation source. The emission decays were detected in the spectral range 475–525 nm selected by a bandpass filter.

### 4.3. Singlet oxygen yield

The singlet oxygen yield of the compound was measured by direct detection of the singlet oxygen emissiton (*λ*_em_ = 1270 nm, detected with a cut-off >1100 nm filter) in aerated acetonitrile against the standard perinapthenone with UV excitation (*λ*_ex_ = 355 nm), according to the method previously described.^26^

### 4.4. Cell culture

Both cell lines HeLa (human cervical cancer) and U2OS (bone osteosarcoma) were purchased from American Type Culture Collection – LGC partnership (Teddington, UK). The cells were cultured in Dulbecco's modified Eagles Medium (DMEM) (Lonza, Cambridge UK) with 10% fetal calf serum (FCS) (Lonza, Cambridge UK) and incubated at 37 °C (5% CO_2_). Cells were routinely checked for mycoplasma contamination.

### 4.5. Cell imaging

Cells were plated at 150 000 cells per well onto sterile coverslips (22 × 22 mm). Following over night incubation, 10 or 20 μM final concentration of 1 (diluted from 1 mM stock solution in DMSO) was added to cells, control wells were incubated with equivalent DMSO concentration and plates were incubated for 5 hours. Following incubation, the cells were washed (3 X PBS) and fixed (4% paraformaldehyde in PBS, 20 min) before being washed (3 X PBS) and mounted to microscope slides (immumount, 20 min). For mitotracker red staining (MitoTracker™ Red CMXRos, Molecular Probes® by Life Technologies Ltd, Paisley, UK) a final staining concentration of 100 nM was added to the cells in 30 min before washing, fixing and mounting. For lysotracker red staining (LysoTracker™ Red DND-99, Molecular Probes® by Life Technologies Ltd, Paisley, UK) a final staining concentration of 75 nM was added for 1 hour before washing, fixing and mounting.

For the multiphoton imaging (Coherent Chameleon femto-second pulsed laser, Inverted Zeiss LSM 510 NLO microscope) 800 nm was used to excite 1; emission was registered in the region 565–615 nm For the co-localisation imaging a confocal microscope was used (Nikon A1) with a 60 × lens (CFI Plan Apochromat VC 60× oil, NA 1.4). A diode laser (405 nm) was used to excite 1 and a sapphire laser (561 nm) was used to excite the co-stains. Pearson's correlation coefficients were calculated using the open source imaging software Fiji (based on ImageJ) and the coloc 2 colocalisation tool. The threshold regression chosen was Bisection.

### 4.6. Proliferation assay – MTT

96-well plates were seeded with cells (HeLa or U2OS at 8000/well) in culture media (DMEM with 10% FCS) and incubated (37 °C, 5% CO_2_, overnight). Treatment solutions were made up in media (DMEM with 10% FCS) with the compound diluted from a DMSO stock (1 mM) to the desired staining concentrations (0, 5, 10 and 20 μM) with the final concentration of DMSO equal in all samples (2%)

### 4.7. Toxicity assay – clonogenic survival

6-well plates were seeded at low density (HeLa, 200 and 400 cells per well) in culture medium (DMEM with 10% FCS) and incubated (37 °C, 5% CO_2_, overnight). Treatment solutions were prepared as above and added (1 mL per well, 5 hours). Following incubation the treatment solution was removed and cells washed (1 X PBS) and fresh media was added (2 mL per well). The plates were left until visible cell colonies had formed. Media was replaced with staining solution (4% methylene blue, 70% methanol, minimum 30 min). The staining solution was washed off and colonies counted with each colony representing a surviving cell.

### 4.8. Transmission electron microscopy

HeLa cells were cultured in T-25 flasks as above until ∼90% confluency is achieved. Media was then removed and the cells washed with sterile PBS (5 mL) before incubation with 20 μM 1 for 4 h. 1 was the removed and the cells washed with sterile PBS (2 × 5 mL). Cells were detached using Trypsin EDTA, washed in media and glutaraldehyde (2.5% in cacodylate buffer) was added to fix the cells overnight at ∼4 °C. Cells were then dehydrated, embedded in Araldite and sectioned in to 85 nm sections and mounted on copper grids before imaging under TEM having either been unstained or stained with standard contrast agent (OsO_4_ 2%, 1 h, UAc_2_ 3%, 25 min, Reynold's Lead Citrate, 5 min). All TEM imaging was carried out using a FEI tecnai 120Kv G2 Biotwin TEM with an Orius SC100 bottom mounted camera using Gatan Digital Micrograph software. Image analysis was performed using imageJ.

## Author contributions

VK, JW and HB conceived and designed the study, RD synthesised and characterised all compounds, LM performed the photo-physical analysis and toxicity assays, LM and JS performed the imaging, RD, LM, JW, JS, VK and HB wrote the manuscript.

## Conflicts of interest

The authors declare no competing financial interests.

## Supplementary Material

RA-008-C8RA00265G-s001
