# Supplementary material for: Pyridazine-bridged cationic diiridium complexes as potential dual-mode bioimaging probes
Source: RSC Adv. 2018 Mar 6;8(18):9670–6. doi: 10.1039/c8ra00265g (PMC6688561; doi:10.1039/c8ra00265g)
Supplement: RA-008-C8RA00265G-s001 [file RA-008-C8RA00265G-s001.pdf]

Electronic Supplementary Material (ESI) for RSC Advances.
This journal is © The Royal Society of Chemistry 2018

Electronic supplementary information

S1: **A:** Normalised UV/Vis and emission spectra for **Complex-1** in acetonitrile and water at ambient temperature in air equilibrated solvent. Normalised excitation λ_{reg} = 600nm) and emission spectra λ_{exc} = 400 nm of **Complex-1** in aerated acetonitrile (dashed lines) and water (solid lines) solutions at room temperature. **B:** Relative initial amplitudes of singlet oxygen luminescence spectra recorded at various laser energy (λ = 355 nm) with Perinapthenone (standard) and **Complex 1** from which singlet oxygen yield of DiIr was determined. **C:** Singlet oxygen luminescence spectra as recorded following excitation of perinapthenone (standard) and **Complex 1** in acetonitrile at 355 nm. Luminescence spectra recorded at 1275 nm. **D:** Relative emission of **Complex 1** in aerated and degassed acetonitrile. Optical densities at excitation wavelength (400 nm) are equal.

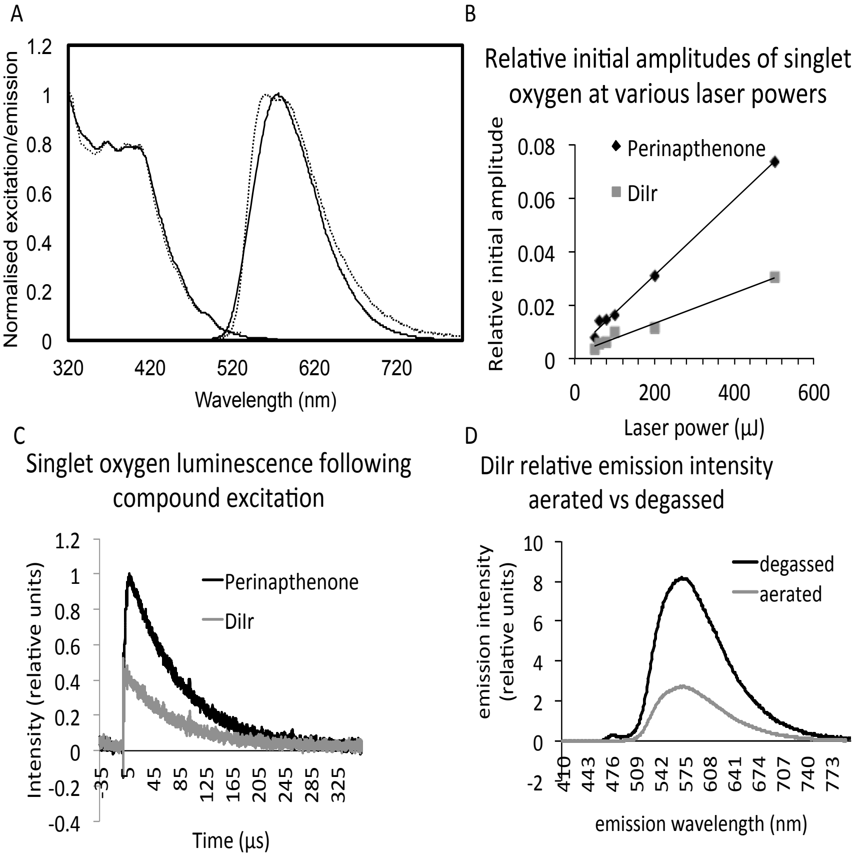

S2: Multiphoton images of **Complex-1** in U2OS cells, **A:** 20 μM, and **B:** 5 μM after 5 hours incubation, emission (left), brightfield (right) and overlay (centre). Excitation at 800 nm and emission 565-615 (green LUT chosen for clarity). Scale bars = 20 μm

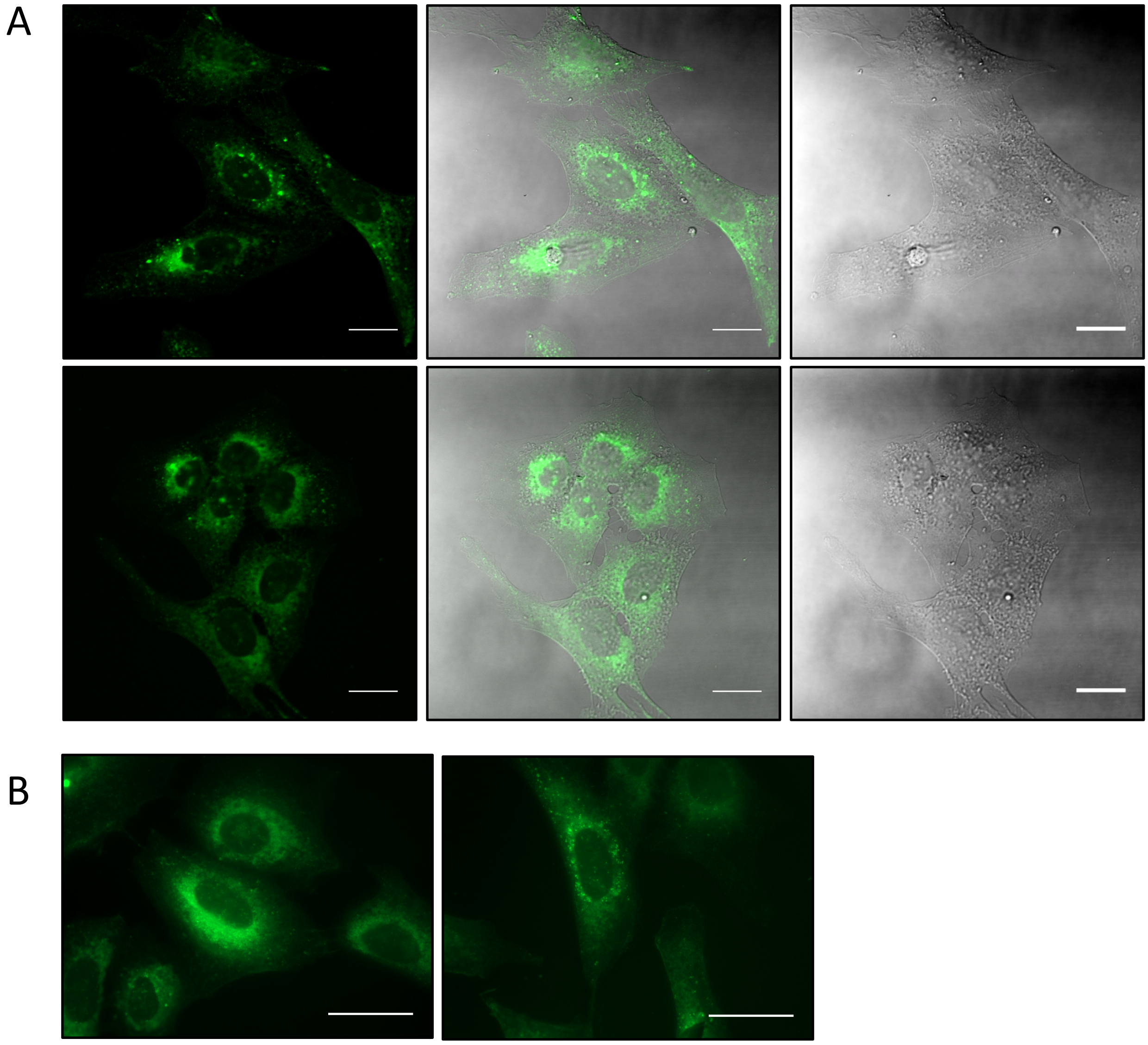

S3: Copies of NMR spectra for all compounds and HRMS for **1**.

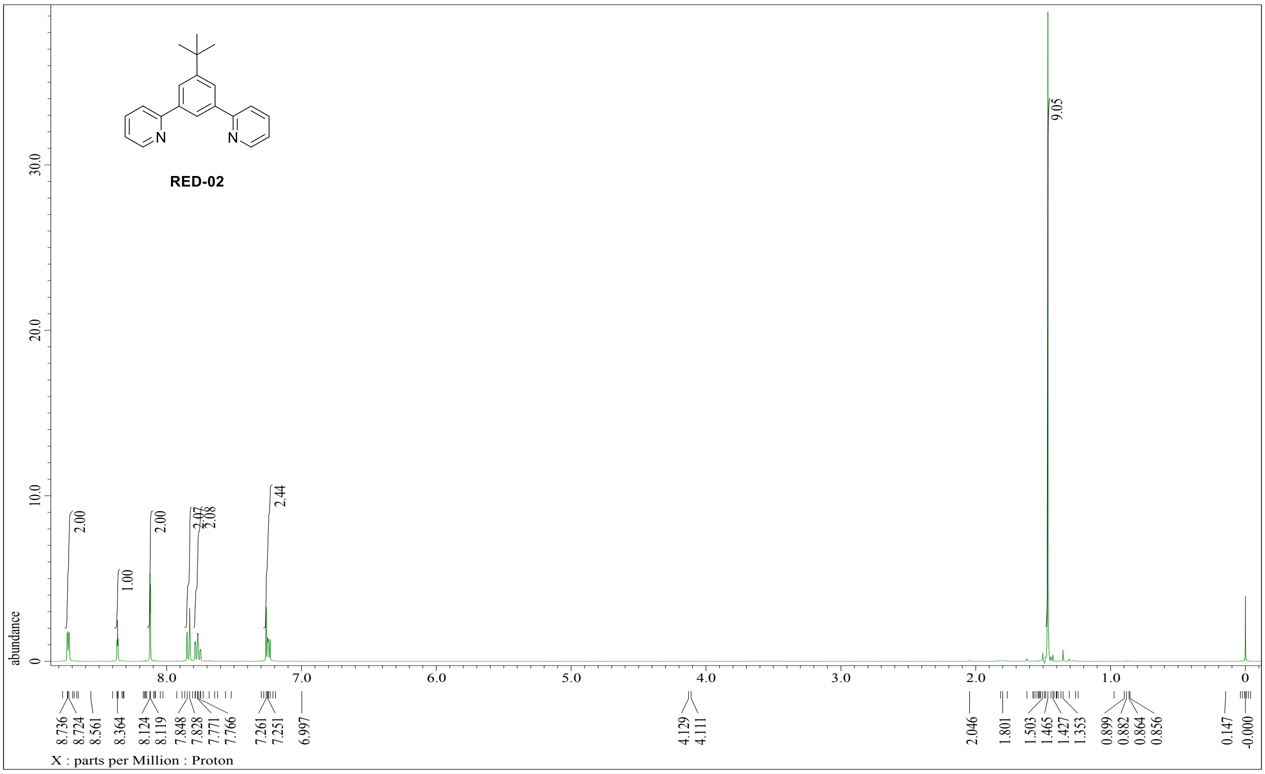

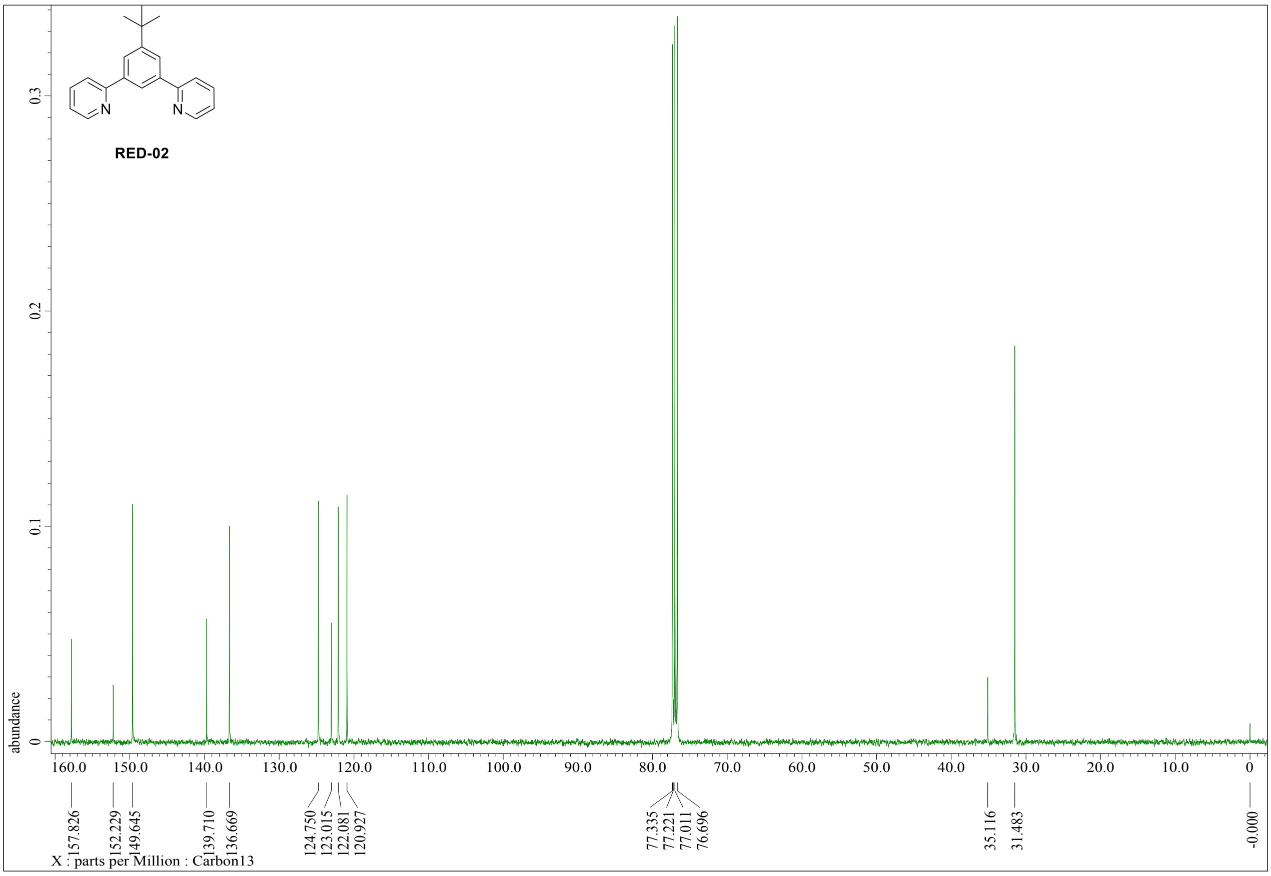

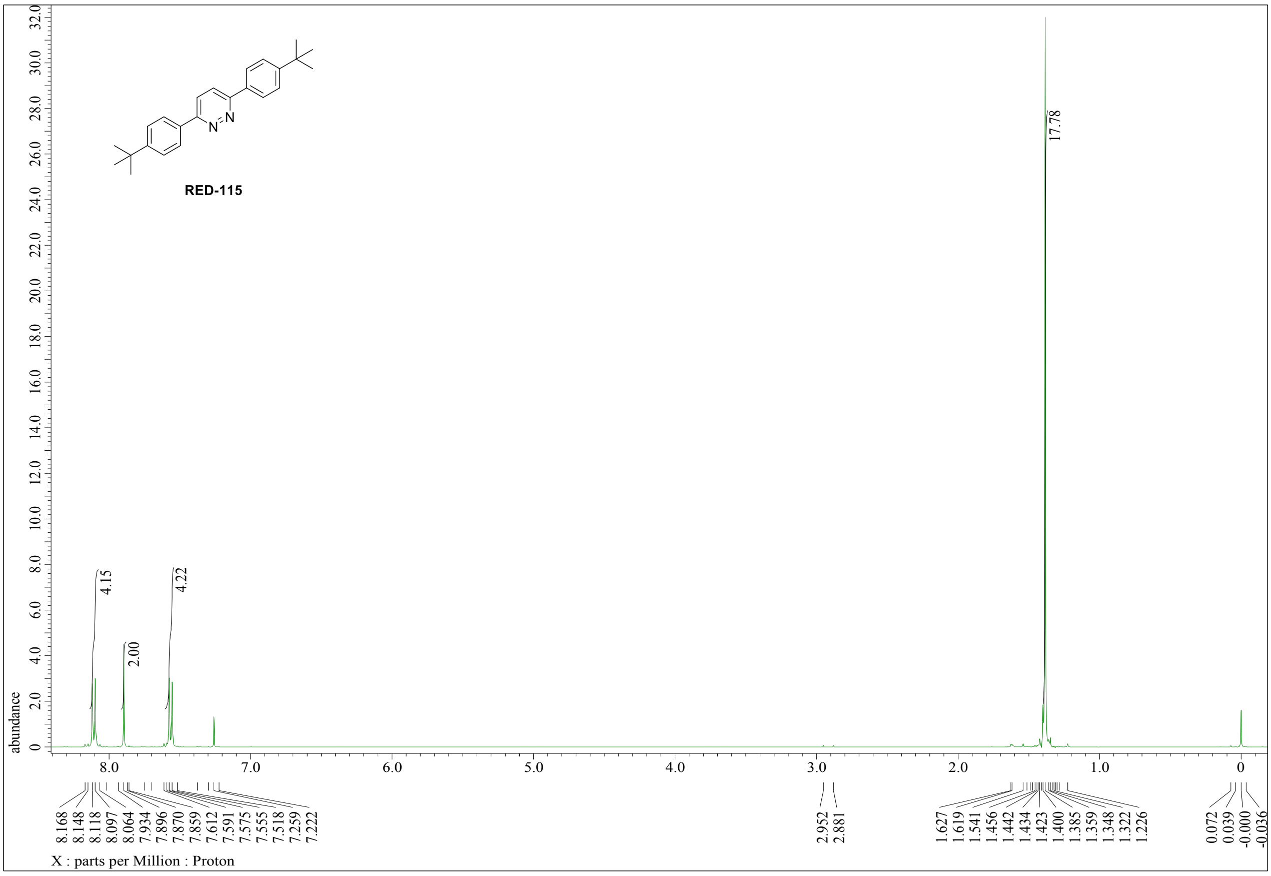
RED-115
abundance
4.15
2.00
4.22
17.78
8.168 8.148 8.118 8.097 8.064 7.934 7.896 7.870 7.859 7.612 7.591 7.575 7.555 7.518 7.259 7.222
2.952 2.881
1.627 1.619 1.541 1.456 1.442 1.434 1.423 1.400 1.385 1.359 1.348 1.322 1.226
0.072 0.039 -0.000 -0.036
X : parts per Million : Proton

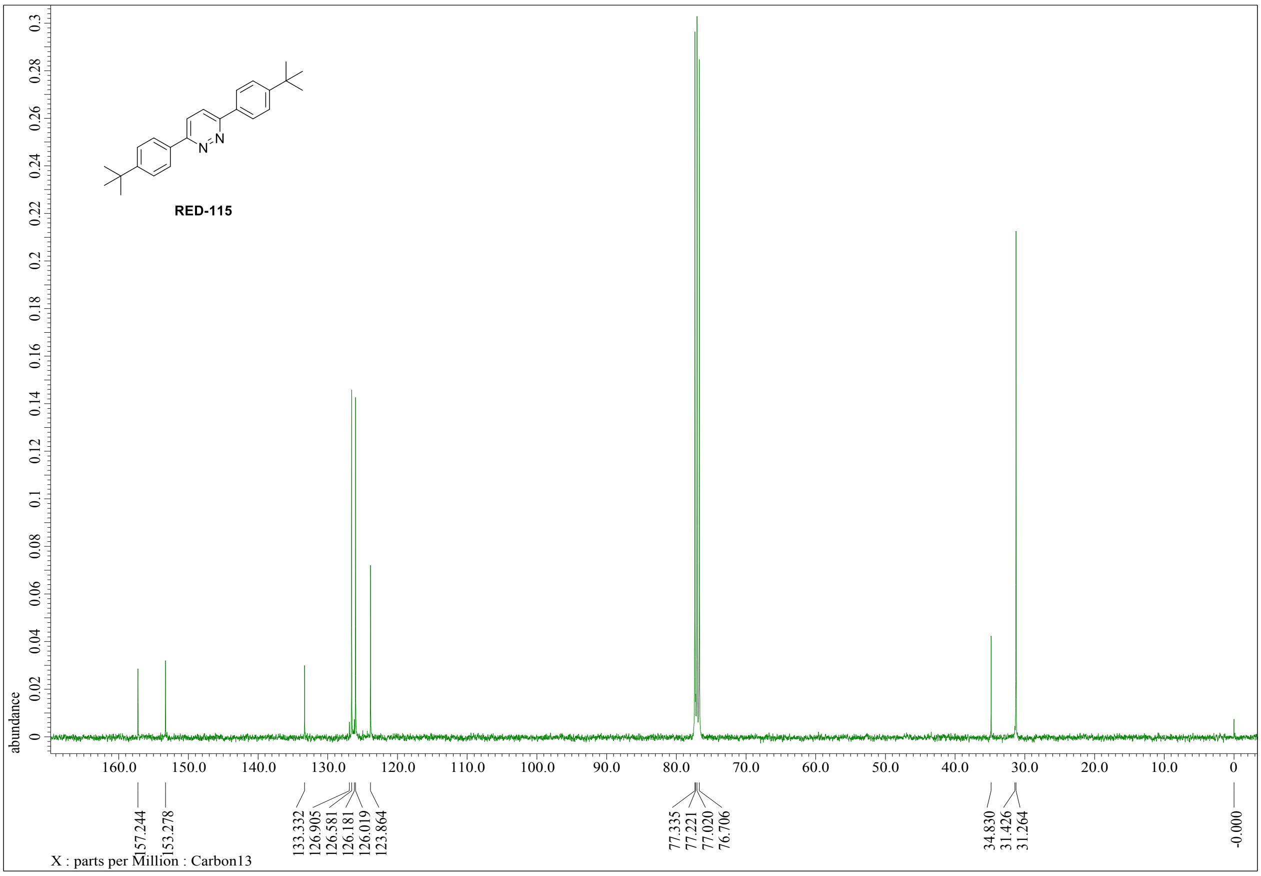
RED-115
abundance
157.244
153.278
133.332 126.905 126.581 126.181 126.019 123.864
77.335 77.221 77.020 76.706
34.830 31.426 31.264
-0.000
X : parts per Million : Carbon13

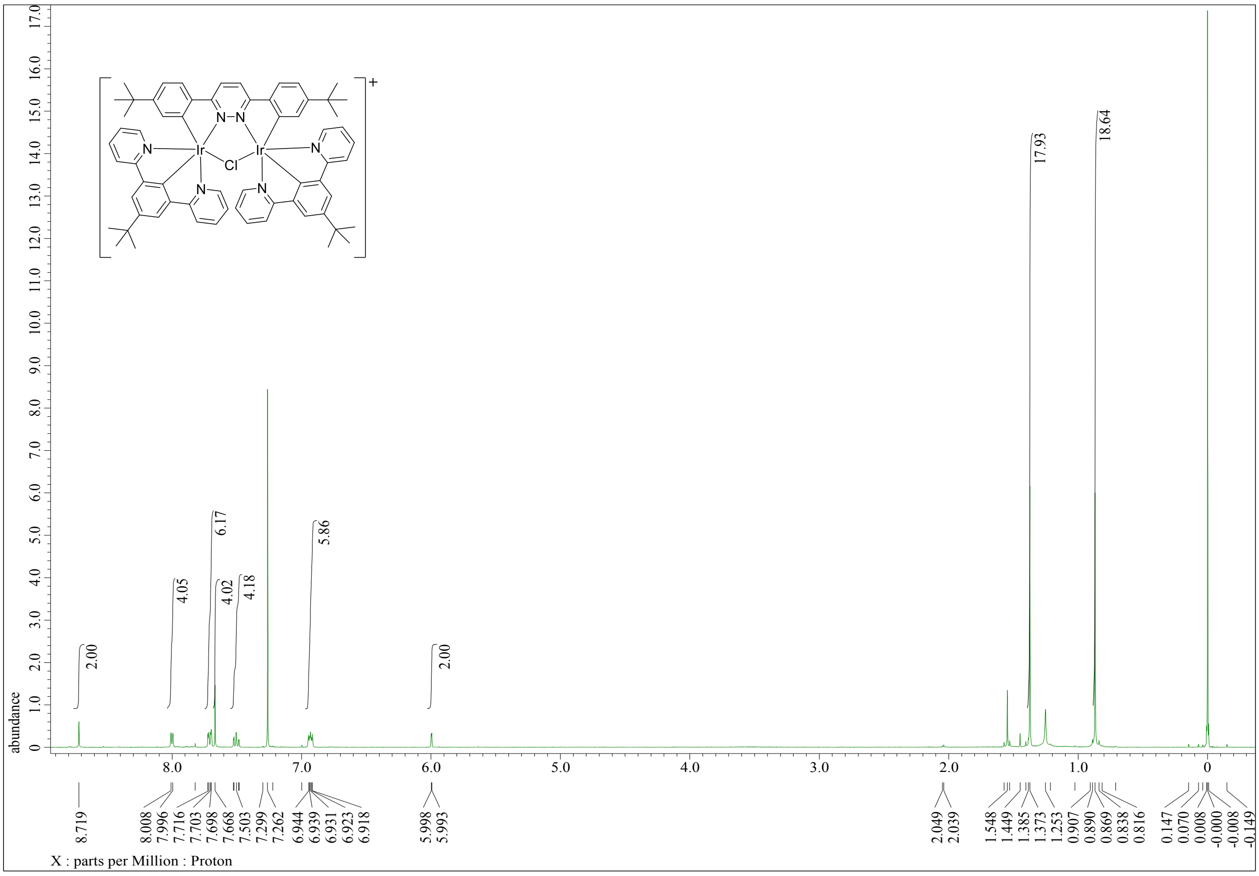
abundance
X : parts per Million : Proton
2.00
4.05
6.17
4.02
4.18
5.86
2.00
17.93
18.64

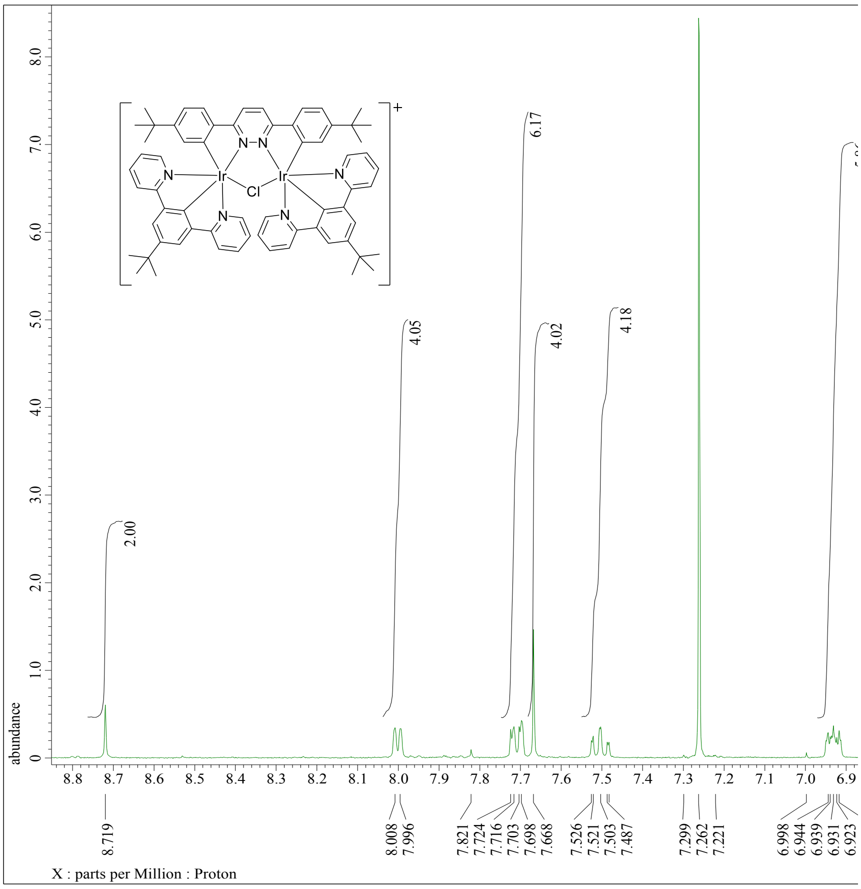
abundance
X : parts per Million : Proton
2.00
4.05
6.17
4.02
4.18

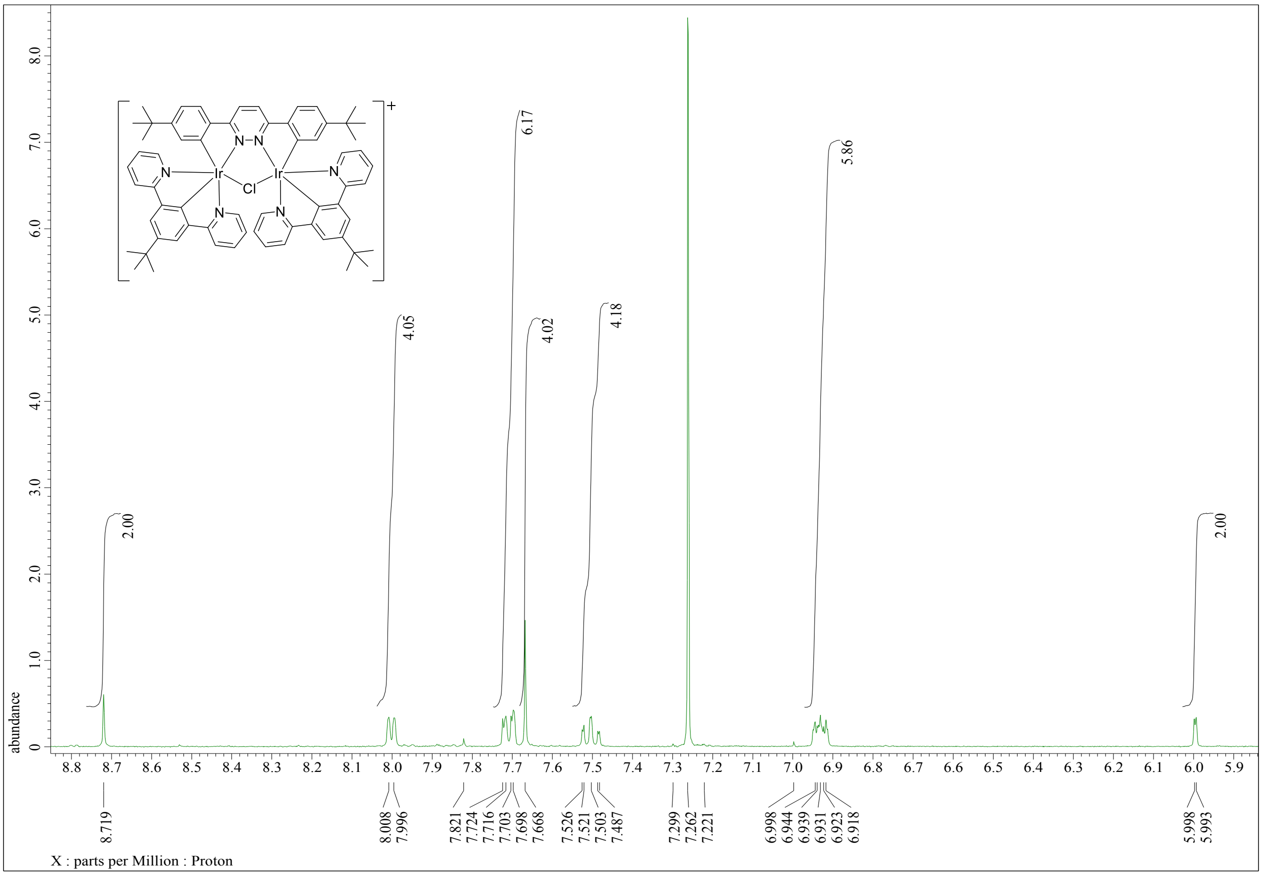
abundance
X : parts per Million : Proton
2.00
4.05
6.17
4.02
4.18
5.86
2.00
8.719
8.008
7.996
7.821
7.724
7.716
7.703
7.698
7.668
7.526
7.521
7.503
7.487
7.299
7.262
7.221
6.998
6.944
6.939
6.931
6.923
6.918
5.998
5.993
N–N
Ir
Cl
N

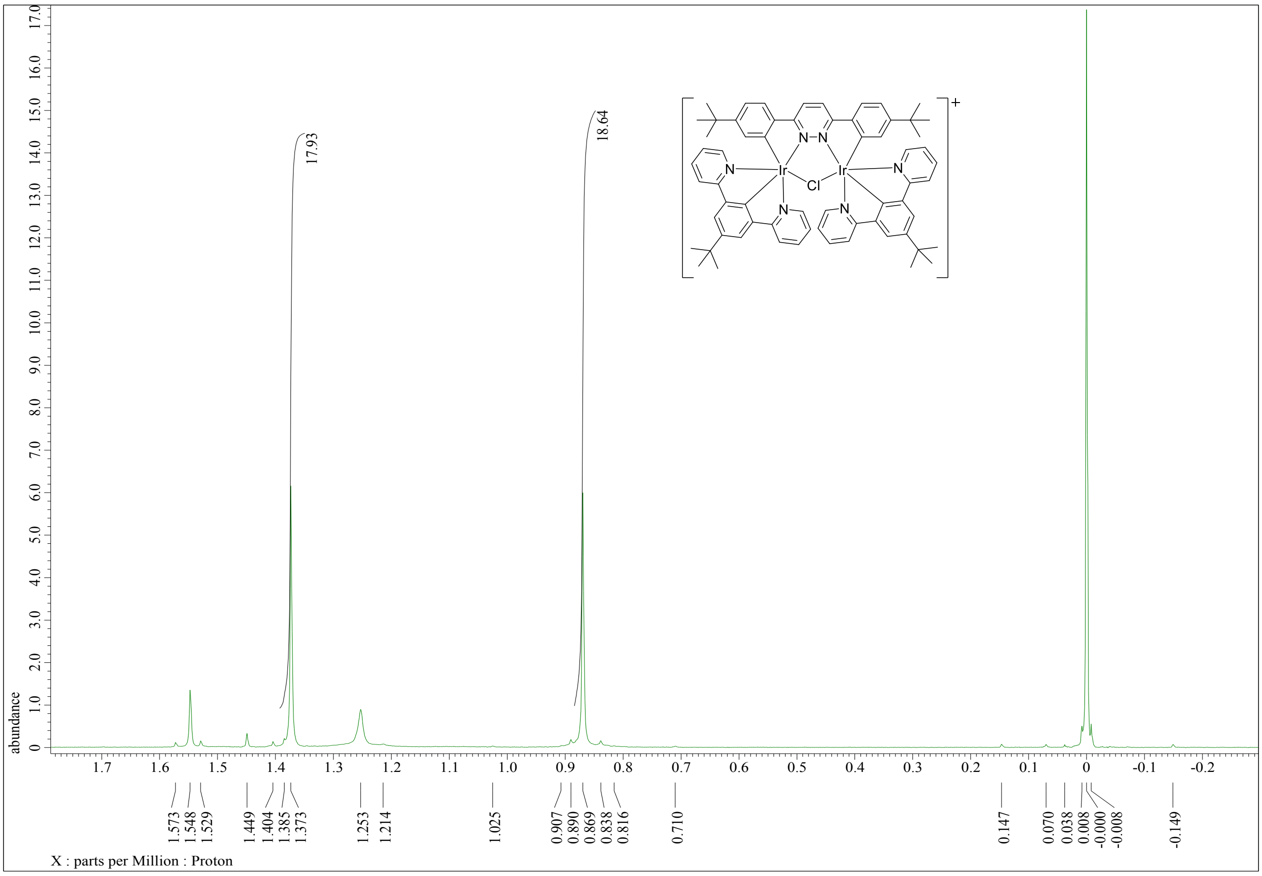
abundance
X : parts per Million : Proton
17.93
18.64
1.573
1.548
1.529
1.449
1.404
1.385
1.373
1.253
1.214
1.025
0.907
0.890
0.869
0.838
0.816
0.710
0.147
0.070
0.038
0.008
-0.000
-0.008
-0.149
N–N
Ir
Cl
N

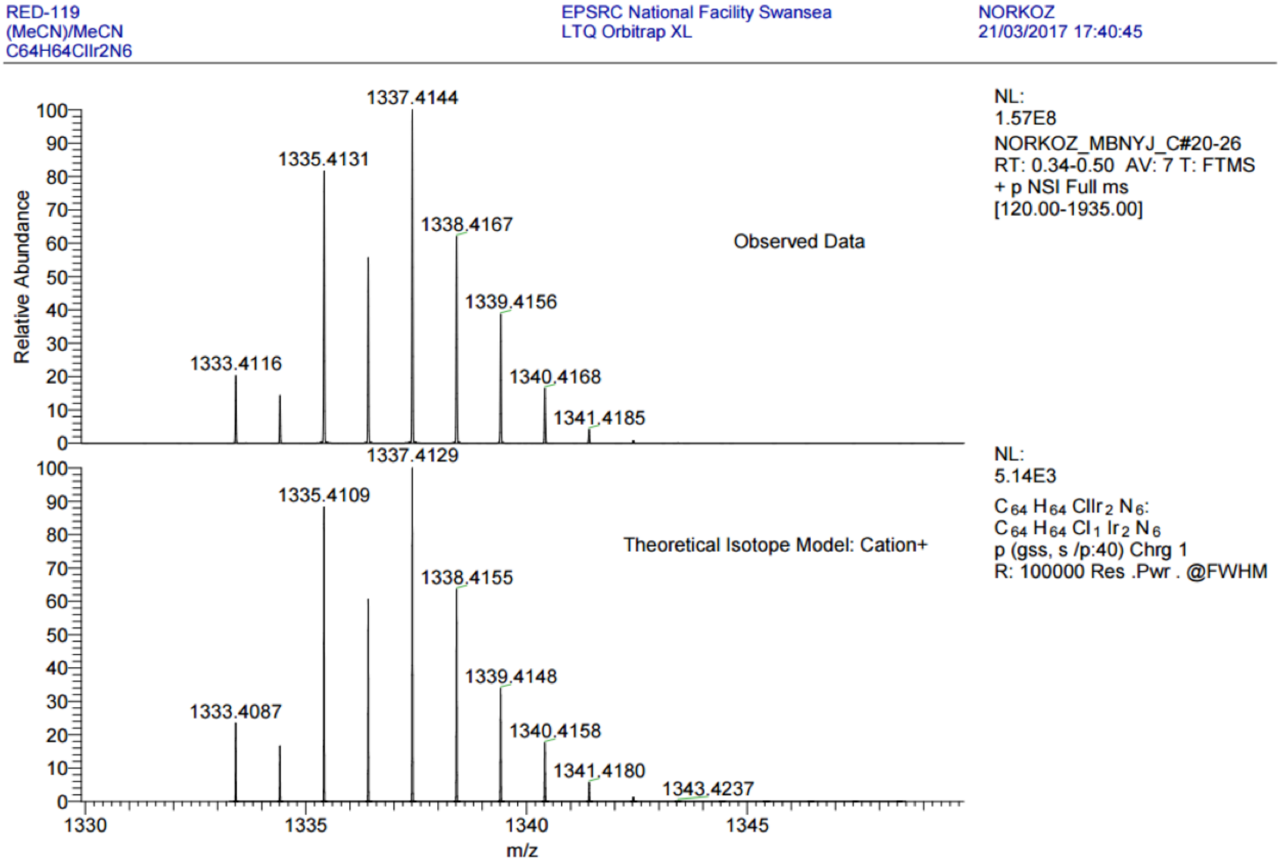

RED-119
(MeCN)/MeCN
C64H64ClIr2N6
EPSRC National Facility Swansea
LTQ Orbitrap XL
NORKOZ
21/03/2017 17:40:45
NL:
1.57E8
NORKOZ_MBNYJ_C#20-26
RT: 0.34-0.50 AV: 7 T: FTMS
+ p NSI Full ms
[120.00-1935.00]
Observed Data
1337.4144
1335.4131
1338.4167
1339.4156
1333.4116
1340.4168
1341.4185
Relative Abundance
NL:
5.14E3
C64 H64 ClIr2 N6:
C64 H64 Cl1 Ir2 N6
p (gss, s /p:40) Chrg 1
R: 100000 Res .Pwr . @FWHM
Theoretical Isotope Model: Cation+
1337.4129
1335.4109
1338.4155
1339.4148
1333.4087
1340.4158
1341.4180
1343.4237
m/z
